# Identification of Nanocellulose Retention Characteristics in Porous Media

**DOI:** 10.3390/nano8070547

**Published:** 2018-07-19

**Authors:** Reidun C. Aadland, Carter J. Dziuba, Ellinor B. Heggset, Kristin Syverud, Ole Torsæter, Torleif Holt, Ian D. Gates, Steven L. Bryant

**Affiliations:** 1 Department of Geoscience and Petroleum, Norwegian University of Science and Technology (NTNU), N-7491 Trondheim, Norway; ole.torsater@ntnu.no; 2 Department of Chemical and Petroleum Engineering, University of Calgary, 2500 University Dr. N. W., Calgary, AB T2K 1N4, Canada; idgates@ucalgary.ca (I.D.G.); steven.bryant@ucalgary.ca (S.L.B.); 3 RISE PFI, N-7491 Trondheim, Norway; ellinor.heggset@rise-pfi.no (E.B.H.); kristin.syverud@rise-pfi.no (K.S.); 4 SINTEF Industry, N-7465 Trondheim, Norway; torleif.holt@sintef.no

**Keywords:** nanocellulose, retention, petroleum, energy, oil, petrochemical, cellulose nanocrystals, nanoparticle

## Abstract

The application of nanotechnology to the petroleum industry has sparked recent interest in increasing oil recovery, while reducing environmental impact. Nanocellulose is an emerging nanoparticle that is derived from trees or waste stream from wood and fiber industries. Thus, it is taken from a renewable and sustainable source, and could therefore serve as a good alternative to current Enhanced Oil Recovery (EOR) technologies. However, before nanocellulose can be applied as an EOR technique, further understanding of its transport behavior and retention in porous media is required. The research documented in this paper examines retention mechanisms that occur during nanocellulose transport. In a series of experiments, nanocellulose particles dispersed in brine were injected into sandpacks and Berea sandstone cores. The resulting retention and permeability reduction were measured. The experimental parameters that were varied include sand grain size, nanocellulose type, salinity, and flow rate. Under low salinity conditions, the dominant retention mechanism was adsorption and when salinity was increased, the dominant retention mechanism shifted towards log-jamming. Retention and permeability reduction increased as grain size decreased, which results from increased straining of nanocellulose aggregates. In addition, each type of nanocellulose was found to have significantly different transport properties. Experiments with Berea sandstone cores indicate that some pore volume was inaccessible to the nanocellulose. As a general trend, the larger the size of aggregates in bulk solution, the greater the observed retention and permeability reduction. Salinity was found to be the most important parameter affecting transport. Increased salinity caused additional aggregation, which led to increased straining and filter cake formation. Higher flow rates were found to reduce retention and permeability reduction. Increased velocity was accompanied by an increase in shear, which is believed to promote breakdown of nanocellulose aggregates.

## 1. Introduction

Chemical flooding with polymers is considered one of the most promising Enhanced Oil Recovery (EOR) methods and has been researched for over 40 years. When polymer flooding is applied to a reservoir, water-soluble polymers are added to the water prior to injection. The main objective is to improve the macroscopic displacement efficiency by increasing the viscosity of the aqueous phase. A higher viscosity of water results in a favorable mobility ratio, which reduces viscous fingering effects and changes the flow pattern in the reservoir [1,2,3].

Today, two main polymers are used commercially; the polysaccharide biopolymer xanthan gum and the synthetic polymer hydrolyzed polyacrylamide (HPAM) [4]. From reported field data for polymer floods, HPAM has been used in about 95% of the projects [5]. China is the leading country in implementation of polymer flooding projects, and several major Chinese oil fields, such as Daqing and Shengli, have applied it as a successful EOR-technique. HPAM is the commonly used polymer in their fields, and from good quality reservoirs it has been reported that the incremental oil recovery has increased by as much as 14% of the original oil in place (OOIP) [5]. Despite these good results, synthetic polymers have limitations. They are sensitive to high reservoir temperatures and susceptible to shear degradation. Even though they develop good viscosities in fresh water, they have poor thickening powers in high salinity. Biopolymers, on the other hand, exhibit great thickening abilities in high salinity water and have good shear stability. However, they are susceptible to microbial degradation in the reservoir, have poor thermal stability and can plug formations. Another drawback with biopolymers is that they have a higher cost than synthetic polymers [2,6]. Many of the polymers used today are considered to be toxic; it is therefore of high importance to find EOR-chemicals that are biodegradable after use.

**Figure 1:**
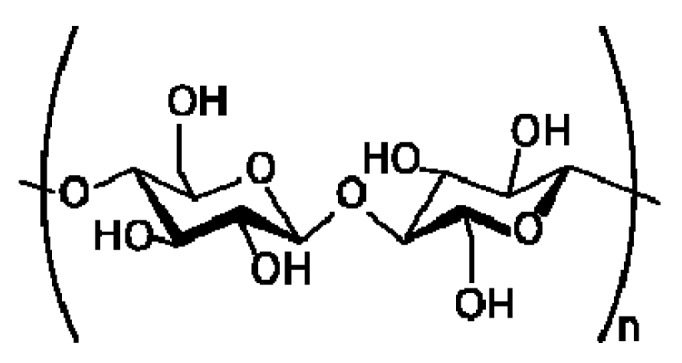
Chemical structure of cellulose. Cellulose is composed of β-1-4-linked d-glucopyranose units.

This paper focuses on the use of novel nanoparticles as an alternative to polymer flooding. These particles are from the nanocellulose family that comprises of cellulosic materials in nanoscale. Cellulose (Figure 1) is a linear polysaccharide polymer that is comprised of many glucose monosaccharide units. There are three free hydroxyl (–OH) groups in each glucose unit in a cellulose chain. These groups govern the important physical properties of cellulose [7]. Cellulose is one of the most important biopolymers on Earth, and it is an abundant resource, as it is the structural component in the cell walls of plants [7]. Wood and cotton are the two most common cellulosic sources.

**Figure 2:**
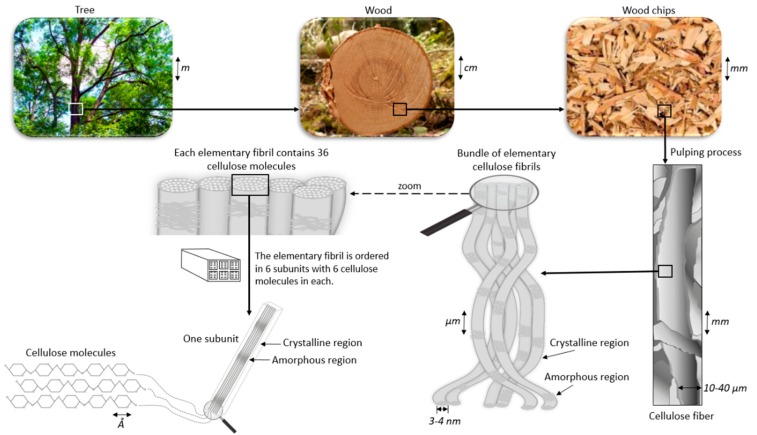
Illustration of nanocellulose. The cellulose fiber consists of crystalline and non-crystalline regions.

Nanoscale cellulose particles may be classified into three main subcategories: bacterial nanocellulose (BNC), cellulose nanofibers (CNF) and cellulose nanocrystals (CNC) [8]. Only the last category is considered in this paper. Cellulose fibers can be converted into CNC by chemical treatment. A cellulose fiber consists of highly ordered (crystalline) and disordered (amorphous) regions (Figure 2). The amorphous regions are removed through acid hydrolysis and only crystalline parts remains [8]. The crystalline parts are in the nanometer size range in all dimensions. CNC from wood is typically ranging from 3 to 5 nm in width and 100 to 200 nm in length [9].

Nanocellulose is an emerging new type of nanoparticle in the petroleum industry and little research exists on its applications for EOR. Preliminary results from oil recovery core floods using CNC particles dispersed in low salinity brine show that the particles have potential as an additive during waterflooding [10]. Experiments were conducted at 60 °C and 90 °C, where the CNC dispersion was injected after water flooding. There was no significant EOR effect during the 60 °C test, but at 90 °C the CNC particles showed a tertiary EOR effect of 3.4% OOIP. A pressure increase with accompanying pressure fluctuations was observed during the CNC injections. Thus, introduction of CNC particles into the porous media affects the fluid flow within the pores. CNC particles could therefore improve microscopic and macroscopic sweep efficiencies through flow diversion [10].

Wei et al. [11] conducted EOR experiments on two types of modified cellulose nanofibrils with different charge densities. Their work showed promising results for nanocellulose as a potential EOR agent. The two types of nanocellulose were injected as a tertiary recovery technique through a heterogeneous micromodel. Improvement of macroscopic sweep efficiency was seen when the nanoparticles were introduced to the system. The nanocellulose with highest charge density had the greatest effect on oil recovery. Interfacial tension (IFT) measurements showed that the particles resulted in a decrease of the dynamic interfacial tension (oil/nanofluid) to the order of 10^−1^ mN/m. A low IFT is a favorable property for an EOR agent. From a microscopic view, it was concluded that the particle with the highest surface charge resulted in a more efficient displacement of the trapped oil in the small pores. Due to the surface activity of the nanofluids, the residual oil was emulsified and entrained in the aqueous phase [11].

Fundamental research concerning particle stability in brine and single-phase flow experiments has also been reported in literature. Molnes et al. [12] established that stable dispersions of CNC were obtained in 1000 ppm NaCl brines. The tested nanocellulose concentrations ranged from 0.5–2.0 wt. %. All dispersions remained stable and the stability was verified through zeta-potential measurements. Furthermore, the CNC dispersion was injectable through a sandstone core. Nevertheless, there were some indications that some of the CNC particles were trapped inside the pore matrix, which is a common phenomenon when dealing with polymers [12]. Another study performed by Molnes et al. [10] tested injection of CNC in Berea sandstone at different temperatures (60, 90 and 120 °C). These results also showed that the particles traversed the core, but some of the particles were retained at the core inlet [10].

**Figure 3:**
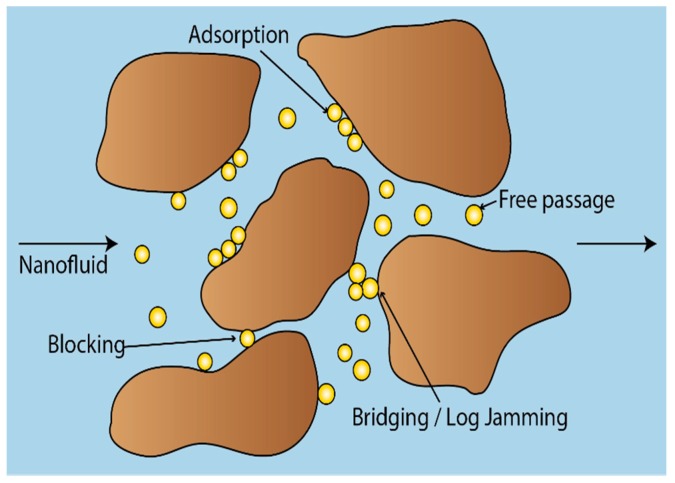
Possible transport outcomes for nanoparticles flowing through porous media.

The aim of the present study was to continue the work on single-phase flow, and identify the main retention mechanism affecting the transport of nanocellulose through porous media. Nanocellulose are not spherical, but rod-shaped particles with an aspect ratio of order of 100:3, which adds a new element of complexity to their flow behavior. During nanoparticle flow through porous media, there are four potential transport outcomes—adsorption, blocking, bridging (log-jamming) or free passage (Figure 3).

Adsorption can occur if the nanoparticle size is much smaller than the pore size and there exists physiochemical interaction between the particle and the pore wall. The particles will then adhere to the pore wall, which could also lead to a wettability change, in addition to loss of matter. This adsorption can be reversible or irreversible.

Blocking can take place if the particle is much larger than the pore throat. If blocking is severe enough, it can result in internal filter cake formation or face plugging. This may be an attractive property in a drilling fluid, as filter cake formation would help minimize fluid exchange with the formation during drilling operations.

Bridging or log-jamming of the pore throat arises when two or more particles with sizes slightly smaller than a pore throat arrive at the pore throat together. Log-jamming may improve sweep efficiency during water flooding. Formation of log-jams in the high permeability channels can divert the flow of subsequent fluids into the unswept low permeability pores, thereby increasing oil recovery.

Free passage happens if the pore throat is large enough for nanoparticles to easily move through [13]. Free passage allows for long-distance transport in the subsurface and is the desired outcome if nanocellulose is to be developed into an alternative to polymer flooding technology.

In general, two opposing effects govern polymer propagation through porous media: retention and inaccessible pore volume (IPV). IPV accelerates polymer propagation, while retention retards it [14]. Polymer molecules can be large compared to some of the pores in a rock. Thus, polymers will not have the ability to flow through all the pore space that is contacted by the brine, giving rise to the concept of inaccessible pore volume in the porous media [15].

Retention is a collective term that involves adsorption and mechanical entrapment (blocking or log-jam). It is an important concern for the EOR application of nanocellulose, as high retention can delay oil displacement and recovery, and will also induce a higher cost as more particles are needed to obtain the desired concentration and viscosity of the injection fluid [14]. Factors influencing polymer retention in porous media include polymer chemistry and composition, formation properties and flow rate. Variables within formation properties include permeability of the rock, clay content, mineralogy, temperature, salinity and pH of the brine. From the formation properties, permeability and clay content seem to have the most effect on retention. Clay has a high specific surface area, thus the retention tends to increase in the presence of clay. Retention also tends to increase for polymers as permeability decreases [1,14].

Polymer retention levels reported in literature range from 9–700 μg/g [15]. According to Lake [5], a good EOR polymer should have retention less than 20 μg/g [5]. If polymer retention values are higher than 200 μg/g, it could have a serious impact on oil displacements rates and the economics of polymer flooding [14]. A study performed by Zhang et al. [16] investigated retention of HPAM through Dundee sandstone (~400 mD) and sandpacks (4.7 D–5.5 D). Two core floods were performed, which resulted in retention values of 16.1 μg/g and 56.5 μg/g. The floodings done in the sandpacks had less retention, ranging from 4.6 to 27.8 μg/g [16]. Martin et al. [17] studied both HPAM and xanthan retention in Berea sandstone cores (350–550 mD) using two different brine concentrations. Seven commercial HPAM polymers exhibited retention values of 15.5 ± 3 μg/g and 25.1 ± 2.1 μg/g in 0.1 wt. % NaCl and 2 wt. % NaCl, respectively. Under the same conditions, three commercial xanthan polymers showed retention values of 7.5 ± 1 μg/g in 0.1 wt. % NaCl, and 11.6 ± 3 μg/g for 2 wt. % NaCl [14,17].

Lotsch et al. [18] measured retention of xanthan in Bentheim sandstones (1600–2000 mD), and got values ranging from 70 to 120 μg/g [18]. This is similar to the values Huh et al. [19] got for injecting xanthan in Berea sandstone cores. They conducted one single-phase experiment where retention was 31 μg/g, and five experiments with two-phase flow, where retention ranged from 49 to 72 μg/g. In their studies they concluded that retention increased with increasing polymer concentration, and it also increased somewhat with the flow velocity [19]. In contrast, sandpack floods with silica nanoparticles showed less retention at higher injection rates, thus implying that nanoparticle retention was not caused by size exclusion, but by physiochemical interactions. In the same study, they also investigated how retention was affected by silica nanoparticle concentration and clay content. They conducted 18 experiments in total and got retention values that ranged from 0.25 to 11 mg/g [20]. Compared to polymer flooding, 11 mg/g is almost 16 times higher than the highest value reported for polymer retention. As nanocellulose is derived from a polymer, but with size in the nanoscale, it is important to determine which factors influence its transport during flow in porous media.

## 2. Materials and Methods

### 2.1. Porous Media

Two types of porous media were used in the transport experiments, and their physical properties are listed in Table 1 and Table 2, respectively. The majority of the experiments were done using unconsolidated sandpacks. The sandpacks consisted of 1-ft-long columns (inner diameter = 1.57 cm) packed with silica sand grains purchased from Sigma-Aldrich (St. Louis, MO, USA).

The other type was core plugs extracted from a Berea sandstone block. The sandstone block was purchased from Berea Sandstone Petroleum Cores (Berea Sandstone Petroleum Cores, Vermilion, OH, USA). These core plugs had an average diameter of 3.8 cm and length of 10 cm. X-ray diffraction (XRD) analyses were performed on five sister samples taken from the same block as the cores. The results show that the sandstone is composed of three main minerals: quartz (93.7 wt. %), microcline (5 wt. %) and diopside (1.3 wt. %).

The absolute permeability was measured by using brine for both media.

### 2.2. Brine

In majority of the retention experiments 0.1 wt. % brine was used, which was prepared using sodium chloride (NaCl). To test the effect of salinity on nanocellulose transport, experiments were also conducted using 0.3 wt. % and 1.0 wt. % brine.

### 2.3. Nanocellulose

**Figure 4:**
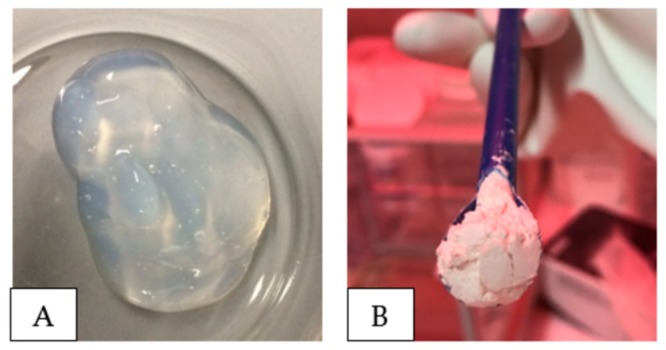
Pictures of nanocellulose stock dispersion (**A**) CNC (USDA) and (**B**) CNC (AITF).

Two main types of nanocellulose were used. One was purchased from the University of Maine. This material was manufactured at the Forest Products Laboratory in Madison, USDA (U.S. Dep. of Agriculture). The cellulose nanocrystals were produced using 64% sulphuric acid to hydrolyze the amorphous regions of the cellulose material, resulting in acid resistant crystals [21]. The acid hydrolysis also results in some surface sulphate groups on the CNC. The stock dispersion is in a gel-form and has a concentration of 12 wt. % (Figure 4A). This CNC is denoted as CNC (USDA) further in this article. The other type of CNC was purchased from Alberta Innovates Technology Futures, and is named CNC (AITF). These cellulose nanocrystals were also prepared by using concentrated sulphuric acid. Both types of CNC tested has a sulphate charge density of ~0.3 mmol/g. The production process for CNC (AITF) has a spray-drying step; thus, the final nanocellulose product is in powder form (Figure 4B).

## 3. Experimental Methods

### 3.1. Atomic-Force Microscopy (AFM)

The microscopic features of the nanocellulose samples were studied by atomic-force microscopy (AFM), using a Bruker Multimode V AFM equipped with a Nanoscope V Controller (Veeco Instruments Inc., Santa Barbara, CA, USA). The instrument was located at the NorFab facility NTNU Nanolab in Trondheim.

The AFM samples consisted of 0.02 wt. % CNC (USDA or AITF) dispersed in either de-ionized water (DIW) or 0.1 wt. % NaCl. A drop of the dispersion was placed on freshly cleaved 10 mm mica (Agar Scientific Ltd., Essex, UK), and was dried using compressed nitrogen gas (N2) before the image could be taken. Images were obtained by ScanAsyst mode in air at ambient conditions. The ScanAsyst-Air AFM tips were provided by Bruker AFM Probes (Bruker Nano Inc., Camarillo, CA, USA). First, one surface picture of the entire sample was taken. From this, a smaller section was chosen to examine in greater detail. The smaller section was usually an area where there was more dispersion between the fibrils, making it easier to study them individually.

### 3.2. Nanocellulose Aggregate Size and Zeta Potential Measurements

The size of nanocellulose aggregates was measured using dynamic light scattering (DLS). With this technique, a monochromatic light beam, such as a laser, shines through the solution. In the solution, particles move randomly due to Brownian motion. The Doppler Effect occurs when the light hits a moving particle and a detector records this change in wavelength of the incoming light. From this, a diffusion coefficient is obtained, which is used in the Stoke-Einstein equation to calculate the hydrodynamic diameter of the particles in solution [22]. This technique is intended for spherical particles, so the measurements done in this study are not exact values of their size. However, the measurements was used to compare the different samples to one another. For each solution, 25 sizing measurements were taken and any measurements with intensity of more than one standard deviation from the median intensity were omitted. The remaining measurements were averaged. This allowed for an accurate representation of the size of aggregates in the bulk solution.

Zeta potential measurements were taken as the average of six measurements. All dynamic light-scattering measurements and zeta potential measurements were performed with a NanoPlus HD—zeta potential and nano particle size analyzer from Particulate Systems (Particulate Systems, Norcross, GA, USA).

### 3.3. Batch Adsorption Experiment

The adsorption experiment was done using 50–70 mesh sand and 140–270 mesh sand. The sand was first rinsed with alternately DIW and 10 wt. % brine, to remove excess silica fines and mimic the purging procedure used in the retention flooding experiments. After the rinses, the sand was placed in an oven to dry.

**Figure 5:**
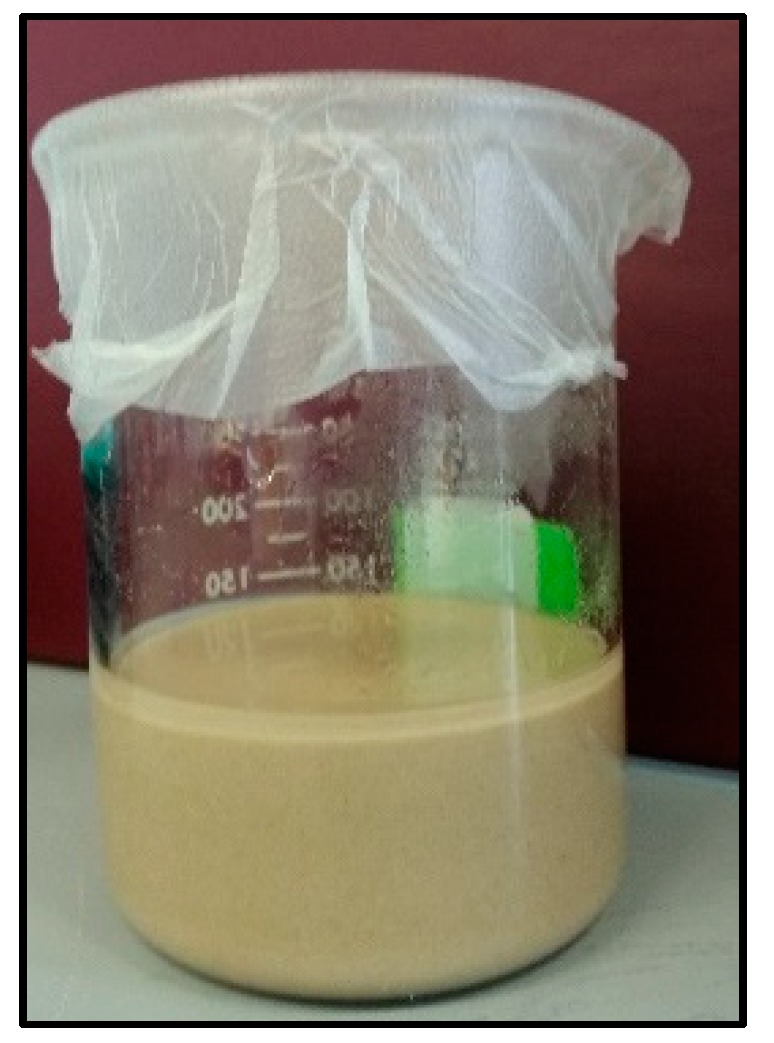
Picture of batch experiment to test for adsorption.

The batch experiment is a static measurement; the setup is illustrated in Figure 5. A beaker was filled with sand and the nanocellulose fluid was poured into the sand beaker until it formed a thin layer above the sand. The beaker was then covered with saran wrap to prevent evaporation. The sand was left to soak in the nanocellulose solution for 48 h; this was considered to be enough time for potential adsorption to occur. A small amount of the nanocellulose solution was kept in a separate beaker to get the initial CNC concentration of the fluid. After 48 h, the nanocellulose solution was filtered from the sand using a mesh. The concentration of the filtered nanocellulose solution was analyzed using phenol-sulfuric acid method (see subsection below), and then compared against the initial concentration of the solution.

The change in concentration from the experiment was compared against the concentration change that would be expected for monolayer adsorption. Theoretical monolayer coverage was estimated by approximating the nanocellulose aggregates as spheres. The diameter of the spheres was taken from the DLS measurements provided in Table 4. The monolayer coverage, per unit of surface area, for a hexagonally packed pattern was then calculated using Equation (1) [23]:

(1) $$\;R_{mono}=\frac{\pi }{3\sqrt{3}}d_{p}\rho_{p}\;$$

(2) $$\;A_{s}=\frac{6}{d_{s}\rho_{s}}\;$$

where *R_mono_* is monolayer coverage (gram CNC/m^2^), *d_p_* is nanocellulose aggregate diameter (m), *ρ_p_* is nanocellulose density (g/m^3^). The retention corresponding to a monolayer of adsorbed CNC in a sandpack is *R_mono_A_s_*, where *A_s_* is surface area per gram of sand (m^2^/g) computed from Equation (2), with *d_s_* the sand grain diameter (m), and *ρ_s_* the sand density (g/m^3^).

#### Phenol-Sulfuric Acid Method

The phenol-sulfuric acid method is used to determine the total carbohydrate content in a sample, as previously described by Dubois et al. [24]. Thus, this procedure was used to measure the nanocellulose concentration from the adsorption experiment. Sulphuric acid breaks down the cellulose into glucose monomers, which are quantified through an absorbance reading measured at 490 nm. The amount of sugar is determined by referencing a standard curve constructed for the particular sugar under investigation (in this case, glucose). One milliliter of sample was added to a glass colorimetric tube, followed by one milliliter of Phenol (5%; Sigma-Aldrich). The mixture was stirred in a vortex mixer, before five milliliter of concentrated sulfuric acid (Romil, Cambridge, UK) was added. When sulfuric acid is added to the solution, the fluid gets a characteristic yellow-orange color due to a reaction between the monomers and the phenol. The tubes were incubated for 10 min and subsequently mixed again in a vortex mixer before they were further incubated for 30 min. A Shimadzu ultraviolet (UV)-spectrophotometer (UV-1800) was used for the absorbance readings at 490 nm, as this is the wavelength for absorbance for hexoses (as glucose). All samples were prepared in duplicates.

### 3.4. Sandpack Retention Flooding Experiments

One experiment took approximately two days to complete, where Day 1 was injection of a tracer fluid and Day 2 was injection of the nanofluid. There were two main procedures for each experiment: a preparation part and the tracer or nano flooding. Four different parameters were tested in these floods: salinity, sand grain size, particle type and velocity (Table 3).

#### 3.4.1. Preparation of Sandpacks

The sand from Sigma Aldrich was rinsed using deionized water (DIW) and high salinity brine (10 wt. % NaCl) over a mesh. It was then dried in an oven before packing. After packing the tube with sand, a vacuum pump was used to remove all the water from the lines in the system. The sandpack was then saturated with DIW. The sandpack was prepared for the tracer- or nano flood by alternating between injection of DIW at a high rate (29 mL/min) and high salinity brine (10 wt. %) at a low rate (2 mL/min). This step removed silica fines that can disturb the UV-visible (Vis) absorbance readings of effluent samples (see next subsection). The final step of the preparation part was to purge the sandpack with brine of the salinity to be used in the nano flood. This purge continued until the UV-Vis signals leveled off.

#### 3.4.2. Sandpack Flooding Procedure

**Figure 6:**
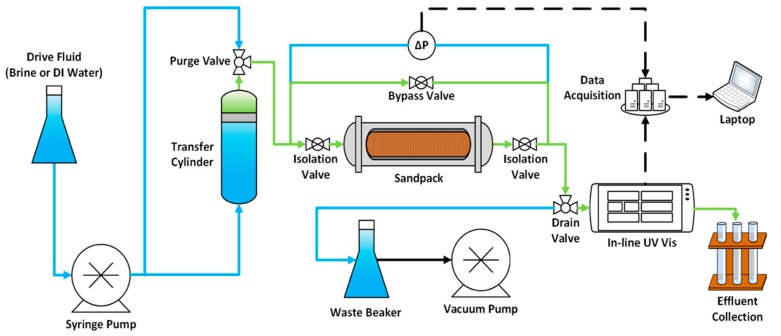
Schematic of the experimental setup used for sandpack retention flood.

Figure 6 shows the experimental setup for the nanocellulose sandpack flooding experiments. An in-line UV-Vis spectrophotometer (DIONEX UltiMate 3400 RS Variable Wavelength Detector from ThermoFisher Scientific, Waltham, MA, USA) was used to calculate the mass balance around the sandpack. This in-line UV-Vis spectrophotometer was found to be an effective way of quantifying nanocellulose concentration. However, the maximum concentration of nanocellulose used in the experiments was capped because the UV-Vis calibration curve became non-linear at high concentrations. The onset of non-linearity varied depending on type of nanocellulose used. The absorbance of both tracer and nanocellulose was measured at a wavelength of 254 nm. Differential pressure was measured during the experiment, which provided information about permeability alterations inside the sandpack.

Sodium iodide (NaI) was used as the tracer because it was shown to behave as a conservative convective-diffusive tracer in a previous study [25]. The tracer flood was carried out with each sandpack before nanocellulose injection, and was performed to characterize the dispersivity in the sandpacks. After each experiment, the tracer breakthrough (BT) curve was compared to the nanocellulose BT-curve. Normally, two pore volumes (PV) of tracer were injected, followed by three PV of brine post flush. Afterwards, the sandpack was prepared for the nanocellulose injection by again alternating between DIW and high salinity brine, as described above. The nanocellulose injection followed the same procedure as the tracer flood, with two PV of nanocellulose injection and then three PV of post flush.

### 3.5. Observing Nanocellulose Retention in Sandpack

Nanocellulose shows significant thermal decomposition at high temperatures. Heggset et al. [26] evaluated the temperature stability of nanocellulose dispersions and found that nanocellulose starts to degrade around 110 °C [26]. The thermal decomposition results in black ash and some char. After each sandpack flood, the sand was emptied onto a tinfoil sheet by applying low-pressure air on the outlet side of the pack, pushing the sand slowly out while approximately maintaining the inlet-to-outlet dimension of the pack. The sand was then baked in a Thermo Scientific HERATHERM Oven at 300 °C to trigger thermal decomposition. Regions of the sandpack containing relatively more nanocellulose would darken more due to the presence of ash and char. This allowed a qualitative assessment of the retention.

### 3.6. Berea Sandstone Retention Flooding Experiments

These experiments were done in a similar manner to the sandpacks. However, the preparation procedure only consisted of the final step, which was to flood the core with the salinity used in the nano flood until a zero-reading on the UV-Vis signal was obtained. Two experiments were run on each core, one tracer flood and one nano flood. CNC (USDA) was the only particle used, and only salinity was varied (Table 3).

#### 3.6.1. Preparation of Berea Sandstone Core Plugs

The cores were rinsed in a soxhlet extraction apparatus with toluene and methanol, and afterwards dried in an oven at 60 °C. The clean and dry cores were then packed in nickel foil before being mounted in the vertical core holder. Each core was then flooded with methanol and afterwards fully saturated with 1.0 wt. % NaCl, which was the brine used in the preparation step of the tracer flood.

#### 3.6.2. Core Flooding Procedure

**Figure 7:**
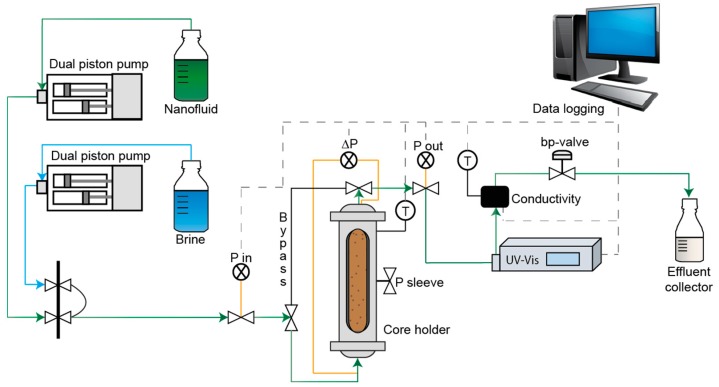
Schematic of the experimental setup used for core plug retention flood.

Figure 7 shows the experimental setup of the retention core flooding. Both experiments were conducted at ambient temperature. The core had a sleeve pressure of 50 bar and the backpressure was kept at 4 bar throughout the experiment. An UV-Vis detector (Azura MWD 2.1 L from Knauer, Berlin, Germany) was used to measure effluent CNC (USDA) absorbance at a wavelength of 254 nm, which in turn was converted to concentration.

A tracer flood was carried out prior to the nano flood. Instead of using NaI as a tracer fluid, the shape of the tracer curve was achieved from conductivity measurements. A conductivity cell was therefore mounted after the UV-Vis detector and it registered the difference in salinity of the produced fluids. The conductivity cell consisted of two platina electrodes and was connected to CDM 83 conductivity meter from Radiometer. The tracer flood consisted of injecting 2.2 PV of 1.2 wt. % NaCl, followed by three PV of 1.0 wt. % NaCl. Afterwards, the core was flooded with 0.1 wt. % NaCl until the UV-VIS signal reached zero. Nanocellulose fluid was then injected for 2.2 PV, followed by three PV of post flush (0.1 wt. % NaCl). Pressures and temperatures were measured and logged throughout the experiments.

### 3.7. Determining Cellulose Retention from Mass Balance

The retention of nanocellulose in the porous media was found through mass balance calculations, by subtracting the produced amount of nanofluid (NF) from the injected amount. The injected amount of particles (*M_inj_*) in mg is given by:

(3) $$\;M_{inj}\left[mg\right]=nPV\;\rho_{NF}\;c\;$$

where *n* is the number of pore volumes of nanofluid that has been injected into the porous media. *PV* is the size of the pore volume in mL, *ρ_NF_* is the density of the nanofluid and *c* is the nanofluid concentration in mg/g.

Prior to the retention experiments, a calibration curve for each tested nanofluid had to be obtained. Different concentrations of CNC (USDA) or CNC (AITF) were injected through the bypass line at a given brine concentration. From this calibration, an equation was obtained for each of the fluids that could convert the UV-Vis signal into CNC concentration (wt. %). After each retention experiment, the CNC concentration was then normalized (${\bar{c}}_{i}$) for each timestep:

(4) $$\;{\bar{c}}_{i}=\frac{\left(c_{i}-c_{min}\right)}{\left(c_{max}-c_{min}\right)}\;$$

where *c_i_* is the produced NF concentration at timestep *i*, *c_min_* and *c_max_* is the minimum and maximum NF concentration during the calibration, which in this case ranged from 0 to 0.5 wt. %, respectively.

By plotting ${\bar{c}}_{i}$ against injected pore volumes, the normalized response curve was obtained. The produced amount of nanofluid can then be found by integrating the area under the effluent response curve using the trapezoidal approximation. The output value for each integrating step (Δ*PV_prod_NF_*) is as below:

(5) $$\;\Delta PV_{prod\_NF}=\frac{1}{2}*\left({\bar{c}}_{i}+{\bar{c}}_{i+1}\right)\left(n_{i+1}-n_{i}\right)\;$$

The produced nanofluid value is given as an equivalent of recovery in pore volumes of dispersion at injected concentration. This means that one can calculate how many PV of nanofluid were produced for each stage (nano flood- or post flush stage). In the experiments, normally 2 PV of NF was injected (for some experiments it was 2.2 PV), followed by 3 pore volumes of brine post flush.

The produced amount in PV of nanofluid during the injection stage (*PV_prod_NF_*) and during the post-flush stage (*PV_prod_PF_*) can be found using Equations (6) and (7), respectively.

(6) $$\;PV_{prod\_NF}=\;\overset{i=2\;PV}{\underset{i=0\;PV}{\sum }}\Delta PV_{prod\_NF}\;$$

(7) $$\;PV_{prod\_PF}=\;\overset{i=5\;PV}{\underset{i=2\;PV}{\sum }}\Delta PV_{prod\_NF}\;$$

Based on the equations above, the produced amount of nanofluid in mg (*M_prod_*) can be calculated:

(8) $$\;M_{prod}=(PV_{prod\_NF}+PV_{prod\_PF})PV\;\rho_{NF}\;c\;$$

The amount of retained particles can then be calculated using Equation (9).

(9) $$\;R_{M}\left[mg\right]=M_{inj}-M_{prod}\;$$

where *R_M_* is the retained mass of particles. *R_M_* can be divided by the dry weight of the porous media (*W_core_* or *W_sand_*) to find the retained mass per gram of rock (*R*):

(10) $$\;R\left[\frac{mg}{g}\right]=\frac{R_{M}}{W_{core}}\;$$

## 4. Results and Discussion

### 4.1. Atomic-Force Microscopy (AFM)

**Figure 8:**
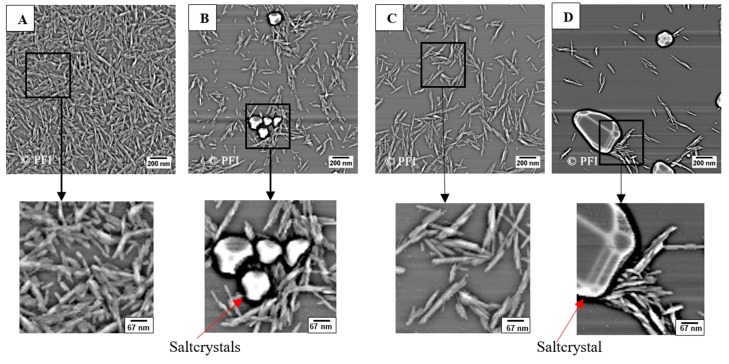
Image (**A**,**B**) is 0.02 wt. % CNC (USDA) in deionized water and 0.1 wt. % NaCl, while image (**C**,**D**) is 0.02 wt. % CNC (AITF) in DIW and 0.1 wt. % NaCl, respectively. The round shapes in image (**B**,**D**) are salt crystals. For all the images, a small section has been enlarged three times to make it easier to see the fibrils and salt crystals in more detail.

The AFM images (Figure 8) show that both CNC types consist of elongated particles. However, from these images, it is not possible to observe if any agglomeration is happening when salt is added to the dispersion. It is important to note that each image is a small selected area of the entire sample. Thus, the amount of crystals on each image is a result of microscope processing. All images were taken after drying with compressed N2. Therefore, if agglomeration happened, it would be difficult to determine if the crystals were partly agglomerated in the dispersion or if the agglomeration happened as a result of the drying method. However, the AFM images show that some of the particles were in the same size range as the DLS measurement (Table 4). Nevertheless, there are also some particles that appear to have sizes that are larger than those found by DLS. This is seen in all images (Figure 8A–D).

### 4.2. Particle Size and Zeta Potential

Table 4 shows the average measured values for the solutions that were used in the retention flooding experiments. The size of the particles in DIW is also included in the table as a reference for the onset of aggregation.

### 4.3. Batch Adsorption Experiments

The concentration measurements from the phenol-sulfuric acid method after 48 h soaking showed little variation from initial values (results not shown). Equations (1) and (2) show that adsorption of even a partial monolayer would have caused substantial change in the aqueous CNC concentration. Thus, adsorption in these tests was too small to be quantified.

### 4.4. Sandpack Flooding Results

This section presents a series of selected breakthrough (BT) curves illustrating the key findings from the sandpack flooding experiments. In each plot, the black dotted curve is the passive tracer. The tracer curve was run prior to each nano flood, and all of the tracer curves overlie each other with minimal variation. The nanocellulose BT-curves were compared against the tracer curve to identify various transport phenomena occurring in the floods. The injection pressure histories for both the tracer- and the nano flood are plotted to show variations of fluid mobility and sandpack permeability.

The influence of particle type, velocity, salinity and grain size on nanocellulose retention are presented in the following subsections. Table 5 summarizes the retention and permeability data for all of the sandpack floods. Full details of the sandpack flooding experiments, including step-by-step protocols, are available online [27].

#### 4.4.1. Effect of Particle Type

**Figure 9:**
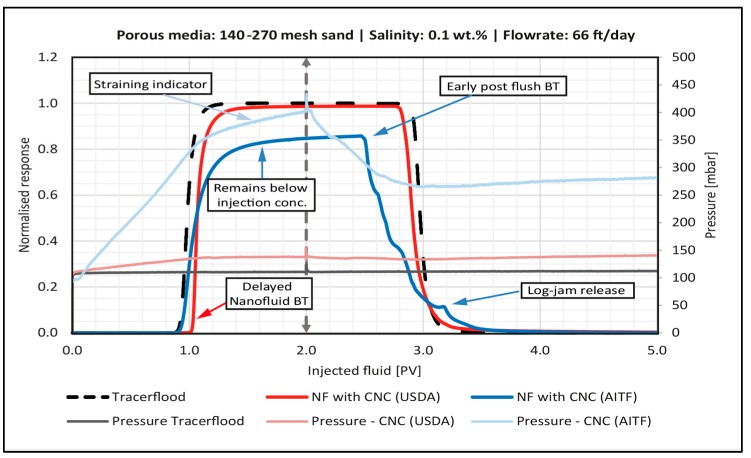
Breakthrough curve for the sandpackflood with 0.5 wt. % CNC (USDA) (red line) and 0.5 wt. % CNC (AITF) blue line. Dashed vertical line illustrates the switch to post flush fluid. The tracer BT-curve is the black dotted line.

The two particle types showed different transport behavior when dispersed at the same salinity. This difference is attributed to their size, summarized in Table 4. In 0.1 wt. % NaCl, the CNC (AITF) exists as aggregates almost five times larger than those of CNC (USDA). The difference in transport properties was most clearly illustrated by the floods in 140–270 mesh sand, Figure 9. The red curve is the flood with CNC (USDA), while the blue curve is with CNC (AITF).

The nanofluid (NF) CNC (USDA) BT-curve showed delayed arrival of the leading edge and early arrival of the trailing compared to the tracer curve. This suggest that adsorption is happening during nanocellulose transport in the sandpack. The effluent concentration also reached the injected concentration between the leading and trailing edges, indicating that an adsorption capacity was reached. The injection pressure increased until one PV was injected, which is consistent with the slightly more viscous nanofluid displacing brine from the sandpack. The injection pressure was steady during the second PV. Combined with the high effluent concentration of CNC (USDA), this suggests that minimal straining or log-jamming was occurring. The retention was 0.20 mg/g-rock (Table 5), which corresponds to 0.11 fraction of a monolayer. This is consistent with the small delay in the BT curve and the small reduction in permeability after the nano flood.

The CNC (AITF) nanofluid BT-curve, which showed less delay in the leading edge, reached less than 90% of the injected concentration, exhibiting early arrival of the trailing edge and an irregular decay, as the post flush continued. Consequently, the retention of CNC (AITF) was much larger at 0.69 mg/g-rock (Table 5), or 7.72% of a monolayer. The injection pressure rose more steeply for CNC (AITF) than for CNC (USDA) during the first PV, consistent with a larger NF viscosity due to larger aggregates of the CNC (AITF). However, the CNC (AITF) injection pressure continued to rise during second PV of NF injection. This indicates that particles are blocking pore throats. Blocking must have contributed to the pressure increase during the first PV as well, and the large reduction in permeability (66%) strongly supports blocking as the mechanism of retention. The early arrival of brine during the post flush is also consistent with pore throats having been blocked during the nanocellulose flood, which reduced the effective pore volume of the pack. The irregularities in the CNC (AITF) NF trailing edge, including a small bump after 3.1 PV, are attributed to retained particles being released from log-jams broken apart during the post flush with brine.

#### 4.4.2. Effect of Flow Velocity

**Figure 10:**
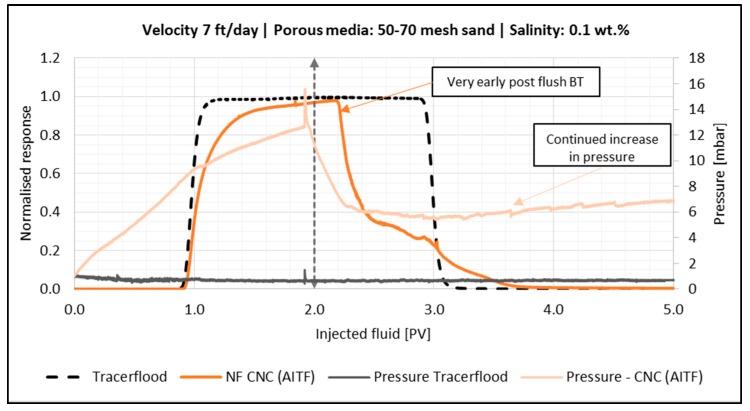
Breakthrough curve for the sandpack flood with 0.5 wt. % CNC (AITF) (dark orange line) using a low velocity (7 ft/day). Dashed vertical line illustrates the switch to post flush fluid. The tracer BT-curve is the black dotted line. The corresponding pressure curves are also shown.

**Figure 11:**
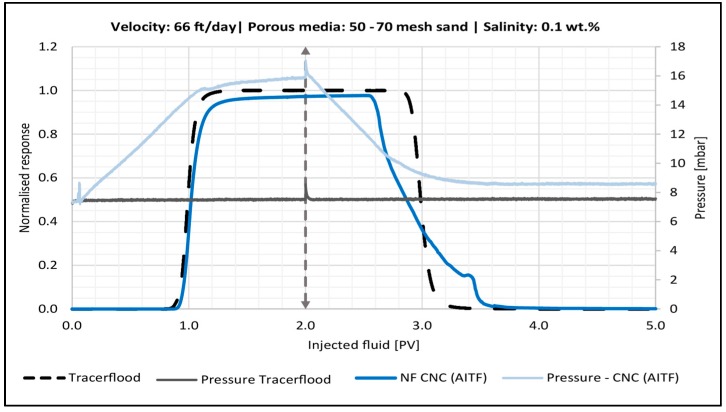
Breakthrough curve for the sandpack flood with 0.5 wt. % CNC (AITF) (dark blue line) using a medium velocity (66 ft/day). Dashed vertical line illustrates the switch to post flush fluid. The tracer BT-curve is the black dotted line. The corresponding pressure curves are also shown.

Several retention mechanisms depend on flow velocity. For example, higher shear rates may increase the formation as well as the breakup of log-jams of particle aggregates. A previous study found that once a critical shear rate is achieved, nanocellulose aggregates will begin to break apart [28]. The effect of flow velocity was only tested for CNC (AITF) as this particle type appeared to be more susceptible to log-jamming. Three velocities were tested in the sandpack experiments: a low (7 ft/day), medium (66 ft/day) and high (521 ft/day). All the experiments were run in 50–70 mesh sand and salinity was kept at 0.1 wt. % NaCl. The BT- and pressure curves for the low and medium velocity can be seen in Figure 10 and Figure 11, respectively.

The low velocity flood had a much-steeper increase in pressure during the second PV of NF injection. The post flush also had an earlier breakthrough compared to the medium velocity experiment, and both floods had some small bumps in the end of the tail of the BT-curve. It is therefore believed that the low velocity leads to more log-jams of aggregated CNC (AITF). The injection pressure during the low velocity post flush was spiky and kept increasing, suggesting a series of break-up/log-jamming events within the retained nanocellulose aggregates.

In contrast to the behavior at lower velocities in Figure 10 and Figure 11, the BT-curve for the high velocity (521 ft/day) had almost the same shape as the tracer, and thus the plot is not shown. Retention and permeability reduction were in the same range as for the medium velocity experiment. The low velocity experiment, on the other hand, showed three times more retention and an order of magnitude of more permeability reduction. This suggests that there exists a critical shear rate, below which retention by log-jamming is severe and above which only minor retention persists after post flush.

The results from these experiments are inconsistent with what has previously been observed with xanthan polymers [19], but follow the same trend as has been seen with silica nanoparticles [20].

#### 4.4.3. Effect of Salinity

Increasing salinity increased the size of particle aggregates (Table 4). Thus, this parameter had the most effect on retention and permeability reduction, since larger particles can more easily block pores. Low salinity solutions were mainly impacted by adsorption, while higher salinity solutions experienced straining and filter cake formation.

**Figure 12:**
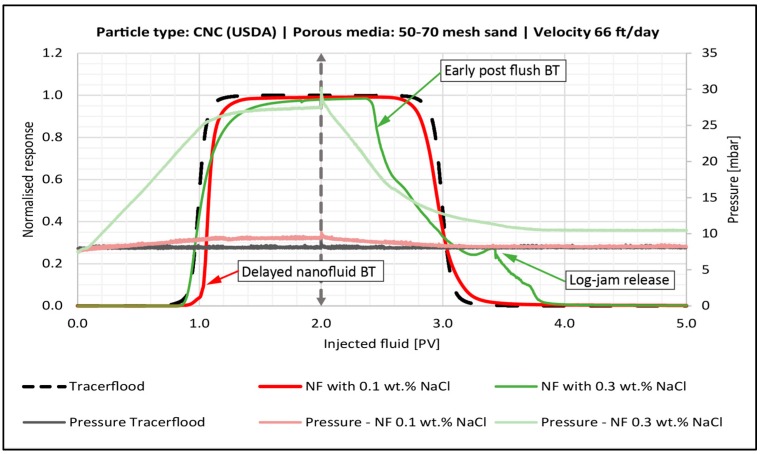
Breakthrough curve for the sandpack flood with 0.5 wt. % CNC (USDA) in 0.1 wt. % NaCl (red line) and 0.3 wt. % NaCl (green line). Dashed vertical line illustrates the switch to post flush fluid. The tracer BT-curve is the black dotted line. The corresponding pressure curves are also shown.

Figure 12 shows the BT-curve for CNC (USDA) in 0.1 wt. % and 0.3 wt. % NaCl. In low salinity, the nanocellulose arrived later than the tracer concentration, then quickly approached injection concentration. This indicates fast adsorption onto a relatively small number of sites in the sandpack, just similar to the finer mesh sand case shown in Figure 9. The pressure drop leveled out during the second pore volume of nanocellulose injection, which indicated minimal straining was occurring. Retention was 0.14 mg/g-rock and permeability reduction was 6% (Table 5), indicating that adsorption was the only mechanism for retention.

For the nano flood with medium salinity (0.3 wt. % NaCl), it did not appear that significant adsorption was occurring because the nanocellulose arrival was no longer delayed relative to the tracer. The pressure slightly increased during the injection of the second PV of nanofluid and the post flush with brine had an early breakthrough. This suggests that log-jamming or blocking of pore throats inside the pack was occurring. Furthermore, the fluid had a smaller volume to traverse, which resulted in early breakthrough during the post flush. The overall permeability reduction using this salinity was 24%, which is over four times higher than what was seen with low salinity.

**Figure 13:**
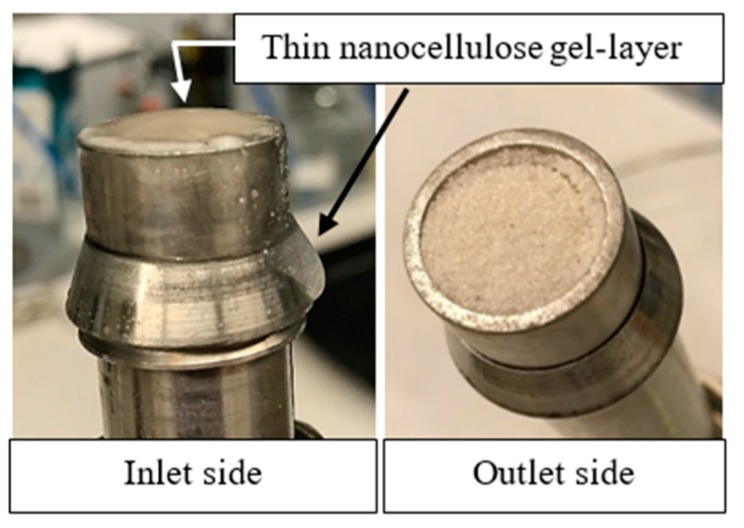
(**Left**) Inlet side of sandpack flooded with 0.5 wt. % CNC (USDA) in 1.0 wt. % NaCl. Thin film of nanocellulose gel sits on the top of the sand. The gel droplet (black arrow) was a result of opening the pack. (**Right**) Outlet side of the same pack after the flood shows no trace of nanocellulose, confirming that filtration prevented transport through the pack.

At the highest salinity (graph not shown), a filter-cake was created at the inlet of the sandpack (Figure 13). This was the limiting case of retention. Large aggregates (see Table 4) formed log-jams in the pores at the face of the sandpack, which then filter out subsequently arriving aggregates. This resulted in 2.2 mg/g-rock retained particles and 98% permeability impairment (Table 5).

For both types of nanocellulose, it was evident that once salinity was high enough, the particle aggregates were large enough for log-jamming to replace adsorption as the primary retention mechanism, and retention and permeability reduction became severe. An increase in retention as a result of higher salinities has also been observed for polymer solutions, such as xanthan and HPAM [14,17].

#### 4.4.4. Effect of Sand Grain Size

The major finding from these experiments was that retention and permeability reduction increased as grain size decreased (Table 5). The effect seemed to be more profound for CNC (AITF) than CNC (USDA), though the same trend was seen for both particles. This is consistent with the conclusions of the previous section that larger aggregates led to log-jamming and greater retention, because the smaller pore throats between smaller grains will likewise encourage log-jamming.

As grain size decreases, the specific surface area increases. If the concentration of adsorption sites for nanocellulose is proportional to the specific surface area, then the extent of adsorption should be smaller in sandpacks with larger grains. The BT-curves for CNC (USDA) support this prediction in the 50–70 mesh sand (Figure 12) and 140–270 mesh sand (Figure 9), where nanoparticle arrival was delayed relative to the tracer curve, while arrival in the 16–30 mesh sand (graph not shown) coincides closely with the tracer curve, indicating minimal adsorption. Confirming this observation, the retention was only 0.05 mg/g-rock and 4.8% permeability impairment.

### 4.5. Direct Observation of Sandpack Retention

**Figure 14:**
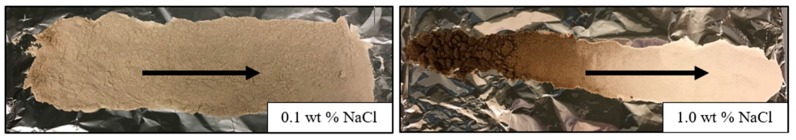
Evidence of nanocellulose retention from post-flood sand baking. Darkness correlates with mass of nanocellulose retained in sand after post flush. Arrows indicate flow direction in original sandpack. (**Left**) 0.5 wt. % CNC (USDA) in 0.1 wt. % NaCl at 66 ft/day resulted in uniformly distributed retention. (**Right**) 0.5 wt. % CNC (USDA) in 1.0 wt. % NaCl at 66 ft/day resulted in large retention near inlet decreasing to little or no retention in the downstream half of the sandpack. Both using 50–70 mesh sand.

Extensive heating of the post-flood sandpacks confirmed that the retention mechanisms inferred from the effluent concentration histories and injection pressure histories described in the previous section. The left image in Figure 14 is for the flood with 0.5 wt. % CNC (USDA) in 0.1 wt. % NaCl. The entire sandpack had darkened slightly and uniformly. This is consistent with a small amount of irreversible adsorption throughout the sand and negligible straining of aggregate log-jams. The latter would be expected to show more retention at the inlet of the sandpack. These findings are consistent with the results from the breakthrough curve shown in Figure 12.

**Figure 15:**
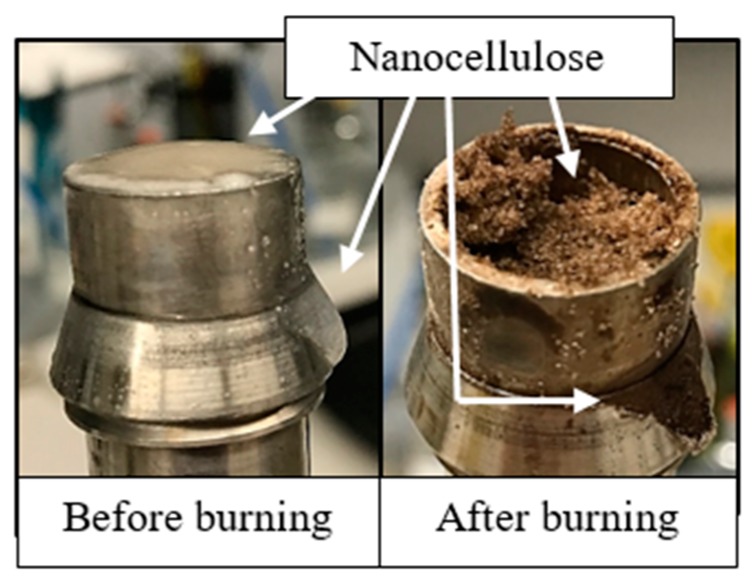
Inlet of sandpack flooded by 0.5 wt. % CNC (USDA) in 1.0 wt. % NaCl. (**Left**) Thin film of nanocellulose gel sits on the top of the sand. (**Right**) Areas containing nanocellulose produced black ash after burning.

The right image is for flood with 0.5 wt. % CNC (USDA) in 1.0 wt. % NaCl. There was a notable relative darkening at the front of the pack, while the outlet seemed to have been unaffected. Thus, nanocellulose retention occurred most heavily at the inlet of pack. This is consistent with a log-jamming leading to straining and filter caking. In addition, this is consistent with the observation of a filter cake forming on the front of the sandpack for this flood. The thin nanocellulose gel-layer that was seen in Figure 13, can also be seen in Figure 15 after heating. From this, it is clear to see that the droplet and film over the sand is not water, but in fact nanocellulose as it has turned dark.

### 4.6. Berea Sandstone Core Flooding Results

This section presents breakthrough curves for two experiments using Berea sandstone core plugs as the porous media. The porous media were more heterogeneous compared to sandpacks and the effect of clay was introduced to the system. The surface charge on the rock might also slightly differ from the sandpack grains. Furthermore, the core plugs have a much lower permeability compared to that of the sandpacks (Table 2).

The data was interpreted in the same manner as for the sandpacks, where the nanocellulose BT-curves are compared against the tracer curve. The pressure curves give further information about permeability reduction and the mode of retention. Salinity was the only parameter varied in these experiments using the CNC (USDA) particles. A summary of the retention and permeability data can be found in Table 6. The permeabilities of the cores was measured before and after the nano flood by flooding brine at four different injection rates.

#### 4.6.1. Effect of 0.1 wt. % NaCl

**Figure 16:**
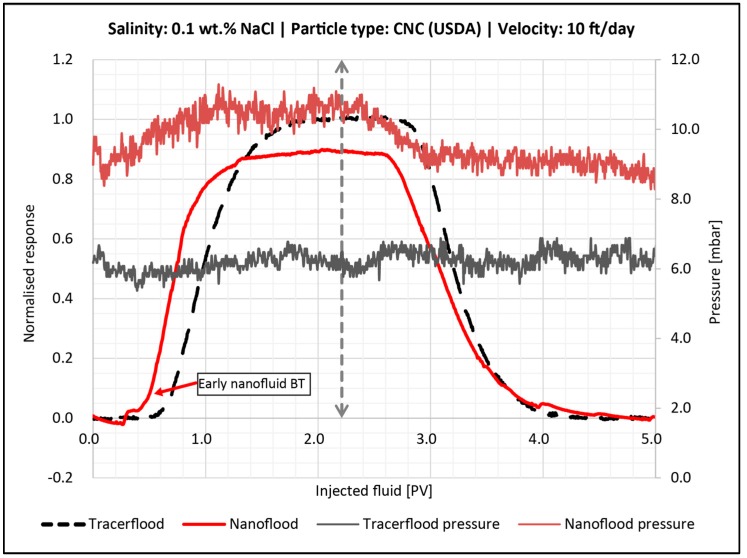
Breakthrough curve for the core flood with 0.5 wt. % CNC (USDA) in 0.1 wt. % NaCl (red line). 2.2 PV were injected followed by 0.1 wt. % brine. The dashed vertical line illustrates when post flush was started. The tracer BT-curve is the black dotted line. The corresponding pressure curves are also shown.

Figure 16 shows that the nanocellulose broke through earlier than the tracer and never reached the injected concentration. The pressure also increased during the injection of the first pore volume, due to injecting of a more viscous fluid. The pressure then stabilized during the second pore volume, and decreased again during the post flush. The total permeability reduction in the core was 18.2%.

The normalized tracer concentration of 0.5 arrived at injection of one PV, while the nanocellulose reached 50% of the normalized concentration at 0.74 PV. The early breakthrough of nanocellulose indicates that some of the PV is inaccessible for this material. The accessible pore volume, which the nanocellulose can flow through, would then be much smaller than the total pore volume of the core. However, it is difficult to accurately determine inaccessible pore volume, as retention of nanomaterial would be an opposing effect for the flow of nanocellulose. Retention would result in a shift for the nanocellulose BT-curve to the right, while IPV shifts the curve to the left. Without an independent measurement of adsorption, it is not possible to determine IPV. Nevertheless, IPV seems to be more profound for the core plugs compared to the sandpacks, where this early nanocellulose fluid BT was not observed. The amount of retained particles at the end of the post flush was quite small—0.015 mg/g rock (Table 6)—as found by mass balances.

#### 4.6.2. Effect of 0.3 wt. % NaCl

**Figure 17:**
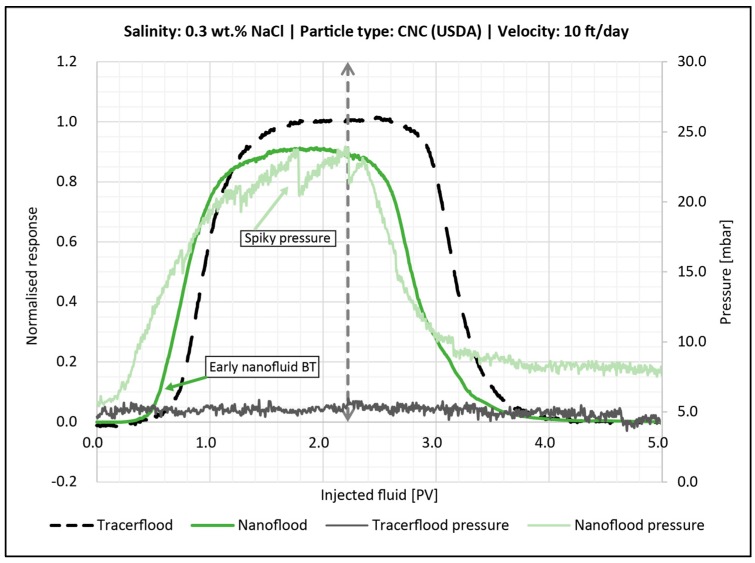
Breakthrough curve for the core flood with 0.5 wt. % CNC (USDA) in 0.3 wt. % NaCl (green line). 2.2 PV were injected followed by 0.3 wt. % brine. The dashed vertical line illustrates when post flush was started. The tracer BT-curve is the black dotted line. The corresponding pressure curves are also shown.

The breakthrough curve for the flood with nanocellulose in 0.3 wt. % brine is seen in Figure 17. The response was similar to the behavior with lower salinity (Figure 16). However, the differential pressure was much higher during this flood, and it was spiky after injection of one PV nanocellulose. Again, there was an early breakthrough of nanocellulose, which is interpreted as a result of inaccessible pore volume to the nanoparticles. As seen in Figure 17 the 0.5 normalized response of nanofluid happens at 0.8 PV (instead of the theoretical 1 PV); the IPV is therefore assumed to be larger than 0.2 PV. Thus, less than 80% of the pore space was available for the nanofluid to propagate through. From the particle size measurements (Table 4), particles in higher salinity had a larger size. Thus, it could be expected that flooding with 0.3 wt. % brine would result in more particles bridging. Particles appeared to log-jam and block off pores, resulting in spiky pressure curves. The peaks of the spikes was interpreted as the failure of a log-jam, after which the remaining particles flowed more easily until they begin to form new bridges and log-jams. This will in turn cause a new pressure build-up. After the 2.2 PV of nanofluid injection, the post flush with brine begun and the pressure decreased.

By mass balance calculation, the retention of particles was determined to be 0.12 mg/g-rock. The overall permeability reduction for this flood was slightly higher than what was seen for the low salinity flood (Table 6).

An interesting finding from the core flood experiments is that the calculated retention values are less than their respective retention values obtained from the sandpack floods. However, for both core plugs and sandpacks at 0.1 wt. % and 0.3 wt. % NaCl, the retention value was less than 200 μg/g.

## 5. Conclusions

In this study, static and dynamic experiments were conducted to investigate nanocellulose retention in porous media. Particle type, porous media, grain size, velocity and salinity were the variables tested for retention. Two types of nanocellulose were used in this study: CNC (USDA) and CNC (AITF). Based on the results, the following conclusions were obtained:

1. In the static adsorption experiments, it was found that neither of the two nanocellulose types seemed to adsorb significantly on the sand grains.
2. CNC (USDA) and CNC (AITF) nanoparticles were different in size, which contributed to different behavior when flooded with 0.1 wt. % NaCl in the sandpacks.
3. Salinity had the largest effect on retention, since it changed the aggregated particle size in the bulk solution. For both porous media (sandpack and core plug), a higher retention was observed when salinity was increased.
4. Retention and permeability reduction in sandpacks increased as the velocity decreased, and the same trend was seen by decreasing the sand grain size.
5. In general, for the sandpack experiments, smaller particles seemed to be dominated by adsorption on sand grains, whereas larger particles were retained in the sandpack by blocking and log-jamming. For the core floods, it also seemed like adsorption was the dominating mechanism in the low salinity flood, while bridging and log-jamming of particles appeared to be the major factor for the medium salinity flood.
6. The sand from the sandpack floods were baked after each experiment, which provided qualitative data and could further support the findings from the calculations and breakthrough curves.
7. In the flow experiments using core plugs, it was evident that the nanocellulose could not enter some of the pore volume.
8. For polymers, 200 μg/g appears to be a maximum value for retention in order for the solution to still be considered viable. By comparing this value against the CNC (USDA) floods in this study, only the flood with 1.0 wt. % NaCl exceeds this criterion where the amount of retained particles was 2.2 mg/g. For CNC (AITF), both the floods with low velocity (7 ft/day) and the flood in the small grain size pack (140–270 mesh) exceeded the maximum retention value. Furthermore, it was not possible to get a mass balance at 0.3 wt. % NaCl for CNC (AITF) due to severe aggregation. Thus, it is likely that the retention value was higher than 200 μg/g for this particle at this salinity.

## Figures and Tables

**Table 1:** Average physical properties of sandpacks.

Sand	Grain Size	Porosity	Permeability	Pore Volume
[Mesh]	[μm]	[%]	[D]	[mL]
140–270	53–105	39	3	23.6
50–70	210–297	37	35	21.9
16–30	595–1190	33	282	19.7

**Table 2:** Physical properties of Berea sandstone cores.

Core	Porosity	Permeability	Pore Volume
[Number]	[%]	[mD]	[mL]
1	16.7	803	19.3
2	16.0	922	18.5

**Table 3:** Variables tested and values selected in retention flooding experiments.

Variable Tested	Values Selected	
	Sandpack Floods	Core Floods
Salinity (wt. %)	0.1	0.1
	0.3	0.3
	1.0	-
Particle Type	CNC (USDA)	CNC (USDA)
	CNC (AITF)	-
Grain Size (mesh)	140–270	n/a
	50–70	
	16–30	
Velocity (ft/day)	7	10
	66	
	521	

**Table 4:** Aggregate sizing data (measured by DLS) and zeta potential data for the solutions used in the floods. The zero salinity value corresponds to the nominal particle size.

Parameter	0.5 wt. % CNC (USDA)				0.5 wt. % CNC (AITF)		
Salinity (wt. %)	0	0.1	0.3	1.0	0	0.1	0.3
Avg. Aggregate Size (nm)	53 ± 3	67 ± 3	298 ± 12	913 ± 132	166 ± 15	321 ± 29	1347 ± 186
Zeta Potential (mv)	-	−30.3 ± 1.8	−21.2 ± 2.4	−11.0 ± 1.3	-	−23.6 ± 2.2	−15.7 ± 1.0

**Table 5:** Retained nanocellulose and permeability data from the sandpack flooding experiments. *, this is the base case experiment. For each parameter varied, the base case value was compared to two other values.

Parameter Changed	Retention [mg/g Rock]		Permeability Reduction [%]	
	CNC (USDA)	CNC (AITF)	CNC (USDA)	CNC (AITF)
**Velocity**				
7 ft/day	-	0.60	-	90
66 ft/day *	0.14	0.15	6	13
521 ft/day	-	0.18	-	8
**Salinity**				
0.1 wt. % NaCl *	0.14	0.15	6	13
0.3 wt. % NaCl	0.19	-	24	97
1.0 wt. % NaCl	2.20	-	97	-
**Grain size**				
140–270 mesh	0.20	0.69	22	66
50–70 mesh *	0.14	0.15	6	13
16–30 mesh	0.05	0.07	5	14

**Table 6:** Retained nanocellulose and permeability data for core flood retention experiments.

Salinity	Retention	Permeability Reduction
	[mg/g Rock]	[%]
0.1 wt. % NaCl	0.015	18
0.3 wt. % NaCl	0.12	19
